# Intraoperative indocyanine green fluorescence for precise resection of nonocclusive mesenteric ischemia: a case report and diagnostic considerations based on pathology findings

**DOI:** 10.1186/s40792-024-02024-3

**Published:** 2024-10-04

**Authors:** Akihito Mizukami, Shinji Furuya, Koichi Takiguchi, Kensuke Shiraishi, Yuki Nakata, Hidenori Akaike, Yoshihiko Kawaguchi, Hidetake Amemiya, Hiromichi Kawaida, Daisuke Ichikawa

**Affiliations:** First Department of Surgery, Faculty of Medicine, University of Yamanashi, 1110 Shimokato, Chuo, Yamanashi 409-3898 Japan

**Keywords:** Nonocclusive mesenteric ischemia, Indocyanine green, Blood flow evaluation

## Abstract

**Background:**

Nonocclusive mesenteric ischemia (NOMI) is characterized by intestinal ischemia caused by spasms in the peripheral intestinal vessels without organic obstruction in the main mesenteric vessels. NOMI can be fatal in case of delayed diagnosis and treatment. Although the use of indocyanine green (ICG) fluorescence in assessing intestinal viability during NOMI surgery is well recognized, there is a paucity of reported cases using this technique. Herein, we present a case of NOMI that was successfully managed through accurate diagnosis and resection of the ischemic intestines guided by ICG fluorescence.

**Case presentation:**

An 81-year-old man presented with abdominal pain. Contrast-enhanced computed tomography revealed intrahepatic portal vein gas, superior mesenteric vein gas, and terminal ileal edema. Considering these findings, the patient was diagnosed with NOMI and emergency surgery was performed. Intestinal edema was observed 30 cm upstream of the terminal ileum without serosal discoloration. ICG fluorescence revealed areas of normal perfusion as well as mild and moderate hypoperfusion. The small bowel, including the hypoperfusion area, was resected. As no clinical signs of residual bowel ischemia were observed during the postoperative course, a second-look operation was deemed unnecessary. Intraoperative ICG fluorescence and histopathological findings indicated mucosal edema in the mildly hypoperfused area and mucosal necrosis in the moderately hypoperfused area.

**Conclusions:**

This case highlights the use of intraoperative ICG fluorescence in the disease. ICG fluorescence is invaluable in assessing the extent of bowel ischemia and guiding precise resection. Thus, future efforts should focus on identifying accumulation of cases and quantification of ICG fluorescence intensity to further improve diagnostic performance.

## Background

Nonocclusive mesenteric ischemia (NOMI) results from bowel ischemia due to spasms of the peripheral bowel vessels without the organic obstruction of the main mesenteric vessels [1]. Owing to its rarity and the lack of characteristic clinical symptoms, NOMI is a diagnostic challenge, often proving fatal because of delayed diagnosis and treatment. Assessing bowel viability is a complex task involved in NOMI surgery; this makes the decision to resect the bowel an inherently challenging one to make.

Surgical management of NOMI is further complicated by the effect of ischemia on bowel viability. Indocyanine green (ICG) fluorescence is emerging as a valuable technique for simplifying this assessment by providing a means to evaluate the degree of intestinal ischemia. Despite its potential, there are few reported cases in which ICG fluorescence is used in the surgical management of NOMI. Herein, we present a case in which the accurate assessment and resection of the ischemic bowel guided by ICG fluorescence proved crucial in saving a patient with NOMI.

## Case presentation

An 81-year-old man presented with sudden onset abdominal pain after dinner and was initially evaluated at a local hospital. Suspicion of NOMI on computed tomography (CT) prompted his emergency transfer to our hospital. Patient had a medical history of angina pectoris, hypertension, diabetes mellitus, dyslipidemia, hyperuricemia, and appendectomy. He was consuming antiplatelet agents (aspirin and prasugrel), antihypertensive drugs, and oral hypoglycemic agents.

On arrival at our hospital, his vital signs were stable, with a blood pressure of 149/79 mmHg, pulse of 78 beats/min, body temperature of 36.6 °C, respiratory rate of 29 breaths/min, and SpO2 of 97% (room air). He was conscious, and had tenderness around the umbilicus, decreased bowel sounds, and a flat and slightly firm abdomen. Arterial blood gas analyses revealed a pH of 7.476, pCO2 of 31.6 mmHg, pO2 of 70.3 mmHg, HCO3 of 23.1 mmHg, base excess of 0.6, and lactate of 2.3 mg/dL (Table 1). Electrocardiogram and echocardiography revealed no notable abnormalities.

**Table 1:** Findings of blood tests at the time of arrival

Complete blood count		Coagulation profile	
WBC	10,940/μL	PT-T	12.2 s
RBC	421 × 10^4^/μL	PT-%	89.3%
Hb	13.2 g/dL	PT-INR	1.06
Plt	21.5 × 10^4^/μL	APTT	29.1 s
		FDP	11.7 μg/mL
Biochemistry		Arterial blood gas analysis (r/a)	
TP	7.4 g/dL	pH	7.476
Alb	3.8 g/dL	pCO2	31.6 mmHg
ChE	21.5 U/L	pO2	70.3 mmHg
T-Bil	0.4 mg/dL	HCO3	23.1 mmol/L
ALP	251 U/L	BE	0.6 mmol/L
γ-GTP	17 U/L	AG	7.7 meq/L
LDH	223 U/L	Na	138 meq/L
AST	23 U/L	K	4.1 meq/L
ALT	21 U/L	Cl	107 meq/L
BUN	25.3 mg/dL	Ca	1.12 meq/L
Cr	0.95 mg/dL	BS	201 mg/dL
eGFR	58 mL/min	lac	2.3 mg/dL
CK	62 U/L	Hb	13.3 g/dL
Amylase	92 U/L		
CRP	0.15 mg/dL		
Na	140 mmol/L		
K	4.1 mmol/L		
Cl	105 mmol/L		
Glu	192 mg/dL		

**Fig. 1:**
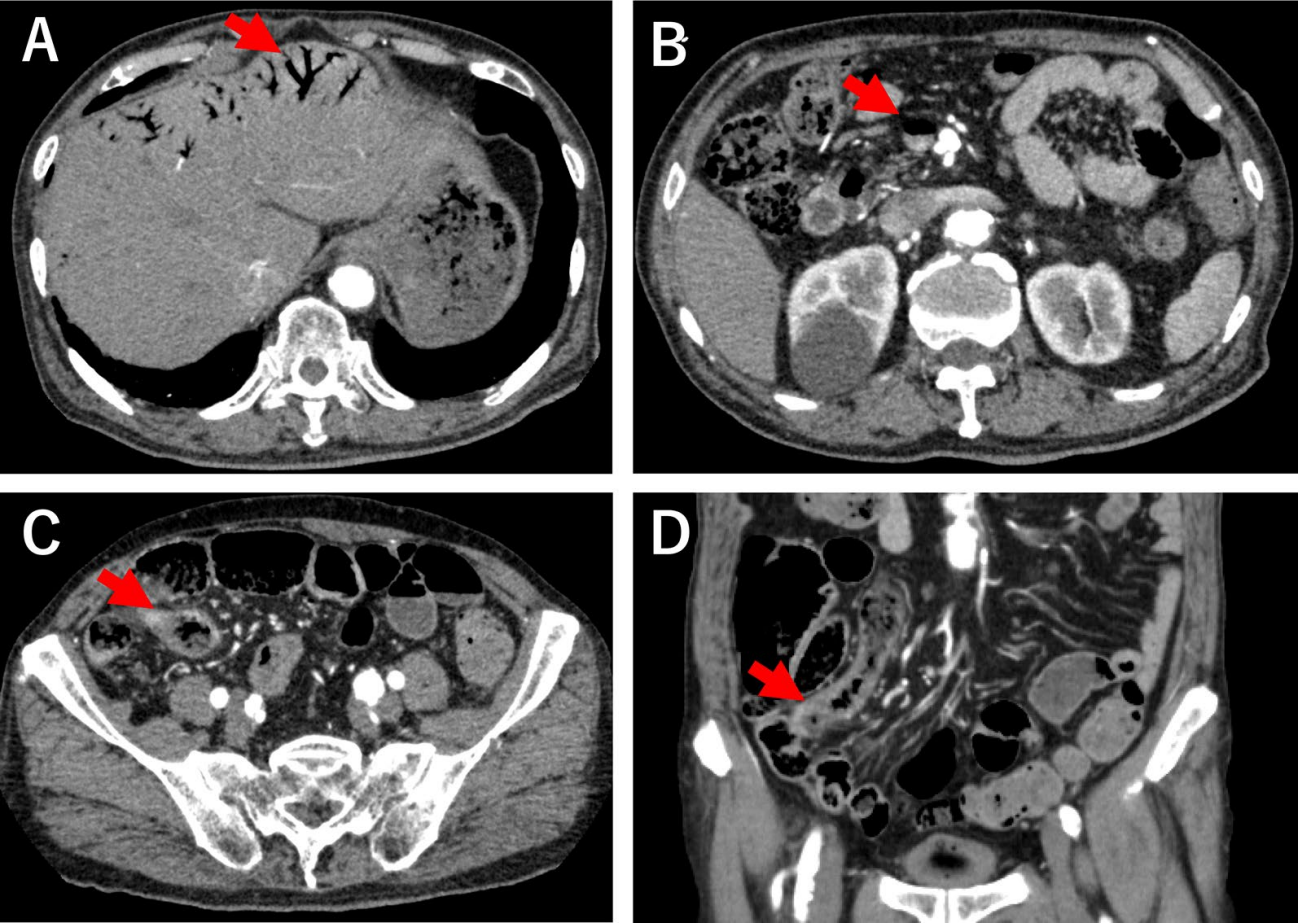
Contrast-enhanced CT. **A** Contrast-enhanced CT of the abdomen revealed intrahepatic portal vein gas (arrows). **B** CT showing superior mesenteric vein gas (arrow). **C**, **D** Terminal ileum is edematous and shows thickening of the wall; however, no obvious contrast effect loss is visible (arrow)

Blood tests revealed mildly elevated white blood cell counts, lactate dehydrogenase levels, and fibrinogen degradation product levels and near-normal C-reactive protein levels (Table 1). Furthermore, contrast-enhanced CT revealed intrahepatic portal vein gas (Fig. 1A), superior mesenteric vein gas (Fig. 1B), and edematous terminal ileum without apparent contrast enhancement (Fig. 1C, D), leading to the diagnosis of NOMI.

**Fig. 2:**
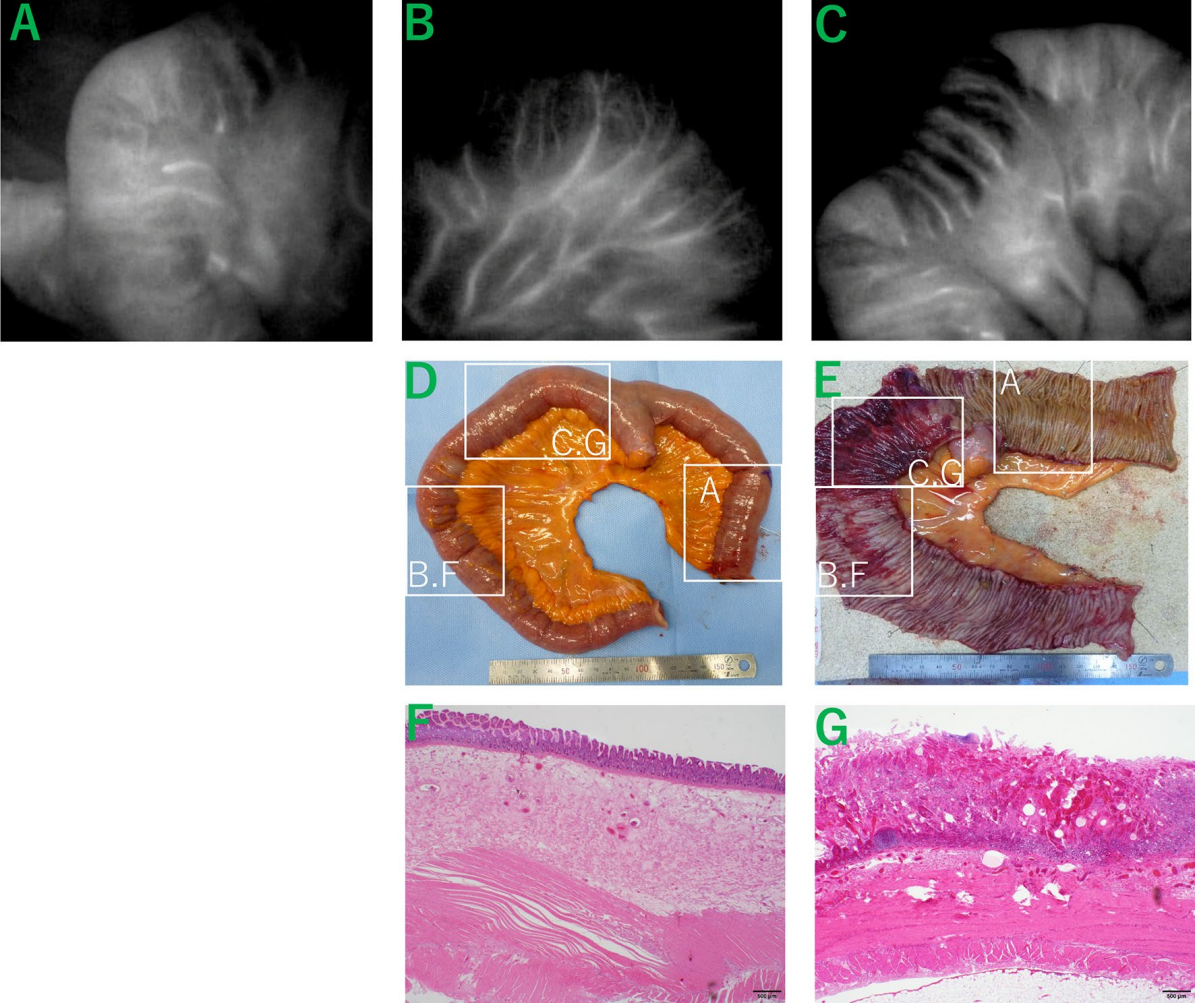
Comparison of Intraoperative ICG fluorescence study, physical examination and histopathological examination. **A** ICG fluorescence test indicating areas of normal blood flow. **B** Areas of mild hypoperfusion on ICG fluorescence testing. **C** Areas of moderate hypoperfusion on ICG fluorescence testing. **D** No color change observed on the serous surface of the excised specimen with the naked eye. **E** Some mucosal areas appear ischemic and necrotic. **F** Area of mild hypoperfusion indicating mucosal edema. **G** Area of moderate hypoperfusion indicating mucosal necrosis

Emergency surgery was performed, and intestinal edema was noted 30 cm upstream of the terminal ileum with no obvious serosal discoloration. Intraoperative ICG fluorescence study (ICG administered intravenously at a dose of 2.5 mg) revealed areas of normal perfusion (Fig. 2A), mild hypoperfusion (Fig. 2B), and moderate hypoperfusion (Fig. 2C) in the edematous bowel. Resection was performed on the small bowel (20 cm to 60 cm from the terminal ileum) where blood flow reduction was observed, anastomosis was performed following the additional resection of a few centimeters of small intestine, which did not present any blood flow issues. A second-look surgery was not considered necessary because there were no clinical signs of residual bowel ischemia during the postoperative course.

The patient’s recovery was uneventful, and he was discharged on postoperative day 9. Although the excised bowel specimen showed no serosal color change (Fig. 2D, E), histopathological examination revealed edematous (Fig. 2F) and necrotic (Fig. 2G) mucosa in areas corresponding to those indicated on intraoperative ICG fluorescence. Specifically, mild hypoperfusion was correlated with mucosal edema (Fig. 2B, F), whereas moderate hypoperfusion was correlated with mucosal necrosis (Fig. 2C, G). In addition, we consistently considered that the diagnosis was NOMI, because of no thrombus or other organic vascular occlusion in the resection specimen. This is a case of the successful use of intraoperative ICG fluorescence to guide NOMI surgery, aiding in precise resection and contributing to a favorable postoperative outcome.

## Discussion

NOMI, first described by Ende in 1958, is characterized by intestinal ischemia and necrosis resulting from circulatory disturbance despite the absence of organic obstruction in the mesenteric artery [1]. This condition arises due to decreased cardiac output and circulating plasma volume, leading to the activation of the renin angiotensin system. This leads to sustained hypoperfusion in the intestine, and sympathetic nerve stimulation causes mesenteric vessel spasms, contributing to the pathogenesis of NOMI [2]. NOMI accounts for approximately 20–30% of the cases of intestinal ischemia and has a mortality rate of 50–80%, mainly due to delayed diagnosis resulting from the lack of clinical symptoms [3, 4]. Furthermore, risk factors for NOMI include cardiac diseases, arteriosclerotic disease, cerebrovascular disease, diabetes mellitus, dialysis, burns, extracorporeal circulation, usage of certain drugs, and advanced age [5, 6]. Recognizing these risk factors and understanding the subtle clinical presentations of NOMI are crucial for early intervention and improved outcomes.

Heer et al. proposed three diagnostic criteria for NOMI: (1) absence of an obstruction in the mesenteric vessel supplying the bowel necrosis area, (2) segmental and discontinuous bowel ischemia and necrosis, and (3) histopathologic evidence of bowel hemorrhage and necrosis [7]. In the present case, although segmental ischemia was not observed, preoperative CT and intraoperative findings supported criteria 1 and 3, which are consistent with the NOMI diagnosis.

The surgical goals include assessing intestinal viability and resecting necrotic portions. Diagnosing gross intestinal necrosis is challenging, with clinical criteria having an accuracy of 57.7%.

In NOMI, where bowel ischemia progresses from the mucosa to all layers, accurate identification of salvageable bowel regions by intraoperative serosal observation [8] is challenging. Although intraoperative mucosal surface observation using a small bowel endoscope has been proposed [9], its practical implementation is complicated and is associated with a risk of mucosal damage. Conversely, the ICG fluorescence method (intravenous ICG solution, Diagnogreen®, Daiichi Sankyo, 2.5 mg/mL) offers a noninvasive and simple diagnostic approach for determining the extent of intestinal necrosis. ICG emits fluorescence under near-infrared light, which is captured by a dedicated charge-coupled device camera, allowing for the visualization of in vivo information, including blood and lymph flow [10]. This method enables the evaluation of the mucosal condition from the serosal surface, providing visualization to approximately 10 mm deeper than the tissue surface [11]. In a study by Ishizuka et al., ICG fluorescence was used to identify bowel necrosis in four of six NOMI cases that were undetectable to the naked eye [12], similar to the present case.

Intraoperative ICG fluorescence shows promise in the diagnosis of bowel necrosis; however, there are notable limitations. First, the package insert mentions shock as a potential side effect, although not significant. Second, the use of Diagnogreen is contraindicated for iodine-sensitive patients because of its iodine content. Third, ICG fluorescence is qualitative and does not quantify organ perfusion, which complicates the determination of the optimal perfusion threshold for bowel preservation [10]. In our case, a retrospective comparison of intraoperative ICG findings with postoperative histopathology enabled us to differentiate between edematous and necrotic intestinal mucosa based on ICG fluorescence intensity. However, it was difficult to determine the preservation potential based on reduced ICG fluorescence intensity alone. Therefore, all areas with decreased fluorescence were resected to avoid potential new ischemia. Fortunately, no postoperative evidence of ischemia in the remaining bowel was noted, thereby obviating the need for a second-look surgery. However, extensive bowel resection may be required in patients with widespread reduction of ICG fluorescence. In cases with severe hypoperfusion in an extensive area, a wide bowel resection cannot be avoided, as it could lead to short bowel syndrome. However, when the mild hypoperfusion area is extensive, determining the extent of resection is difficult. The basic flow is that the mild hypoperfusion area is preserved and re-evaluated at the second-look operation. However, if the patient’s general condition makes it difficult to perform a second-look operation, it may be necessary to resect the mild hypoperfusion area at the first surgery in preparation for short bowel syndrome. When the mild hypoperfusion area is not extensive, it may be possible to avoid the second-look operation by resecting the entire hypoperfusion area, as in this case. A PubMed search using NOMI and ICG as keywords revealed five cases, including this case. Three cases were anastomosed at the primary surgery, and the second look operation could be omitted (Table 2) [13–16]. According to Ishizuka et al., 83% was performed ICG test had no anastomotic intestinal problems [12]. Using ICG potentially led to this result because it can detect the ongoing ischemia with only ischemia on the mucosal surface and no change on the serosal surface. If ICG test is performed, the possibility of reischemia is low [12]. Moreover if all areas of reduced blood flow can be resected, a primary anastomosis may be considered. However, if the cause of hypoperfusion of the intestinal tract is clear and has not been corrected before surgery, or if the area showing ischemia on ICG examination is extensive and the resection of the entire area would result in short bowel syndrome, we should opt for second-look operation. In addition, it is possible that interpretations of the ICG study may differ because of different experience, however, we believe that such bias can be corrected by quantifying the ICG test. Thus, it is essential to gain experience by comparing intraoperative ICG fluorescence intensities with pathological findings.

**Table 2:** Past case reports of NOMI treatment with ICG test

No	Author	Year	Age (y)	Sex	Change of the serosal surface	Areas of reduced blood flow that can be identified only by ICG test	Degree of decrease in ICG fluorescence	Bowel resection	Anastomosis	Second look operation	Pathological findings	Outcome	Ref
					first surgery/ second surgery		first surgery/ second surgery	first surgery/ second surgery					
1	Nitori	2014	84	M	Yes/ –	No	severe/ –	Yes/ –	first surgery	No	transmural ischemic necrosis	transfer	[13]
2	Irie	2017	62	M	Yes/ No	No	unknown/ not done	No/ No	–	Yes	–	discharge	[14]
3	Nakagawa	2018	89	M	No/ –	Yes	moderate/ –	Yes/ –	first surgery	No	mucosal necrosis	transfer	[15]
4	Miyashita	2023	47	F	Yes/ –	No	moderate/ –	No/ –	–	No	–	Reoperation	[16]
5(Our case)	Mizukami		81	M	No/ –	Yes	mild ~ moderate/ –	Yes/ –	first surgery	No	mucosal edema ~ necrosis	discharge	

## Conclusion

We experienced a case of NOMI in which intraoperative ICG fluorescence was instrumental for a swift diagnosis. To determine the extent of bowel resection, it is important to evaluate not only the gross findings but also the area of bowel ischemia using ICG fluorescence. Moreover it is necessary to accumulate cases and quantify the intensity of ICG fluorescence to further improve and establish appropriate diagnostic criteria.

## Data Availability

Not applicable.

## List of Abbreviations

NOMI: Non-occlusive mesenteric ischemia
ICG: Indocyanine green
CT: Computed tomography
MDCT: Multi-detector computed tomography
